# Editorial: A large-scale biology view of crop-environment interaction: the influence of water and temperature stresses on the development of cereal and horticultural crops

**DOI:** 10.3389/fpls.2023.1235466

**Published:** 2023-06-22

**Authors:** Sowbiya Muneer, Keting Chen

**Affiliations:** ^1^ Horticulture and Molecular Physiology Lab, School of Agricultural Innovations and Advanced Learning, Vellore Institute of Technology, Vellore, Tamil Nadu, India; ^2^ Genetics, Development, and Cell Biology, College of Agricultural and Life Sciences, Iowa State University, Ames, IA, United States

**Keywords:** crop environment interaction, metabolomics, proteomics, transcriptomics, temperature stress, water/drought stress

Temperature is an essential factor that plays a role in the expansion and maturation of plants; when the temperature is either too high or too low, the plant’s output, in terms of both quantity and quality, is reduced (Wahid et al., 2007; Liu et al., 2013; Zhu et al., 2013). As a direct result of global warming, we are seeing an increase in the frequency of extreme temperature fluctuations (IPCC, 2018). It has been shown that high temperatures can increase transpiration (for cooling purposes in the presence of soil water) and mitochondrial respiration that occur when temperatures are elevated to 1.5 degrees Celsius above their pre-industrial levels is one of the negative effects of high temperatures. The strain caused by high temperatures also has an effect on cellular, physiological, biochemical, and molecular responses. For example, protein denaturation and aggregation, disruption of cellular homeostasis, and an increase in fluidity in lipid membranes are some of the effects of this stress. Another negative effect of high temperatures is that enzymes in chloroplasts and mitochondria become inactive as a result (Howarth, 2005; Barnabás et al., 2008; Li et al., 2014; Razi et al., 2021). This damage ultimately leads to the formation of reactive oxygen species (ROS), which have the potential to cause harm to biological molecules (Razi and Muneer, 2021; Sahithi et al., 2021). Reprogramming their transcriptome, proteome, and metabolome is how plants react when under the stress of an altered cellular metabolism brought on by high temperatures. Because of these shifts, plant adapts to a new metabolic equilibrium even when subjected to high temperatures. It is common knowledge that temperature has a significant impact on the growth and development of plants. As a result of this well-known fact, agriculturalists and horticulturists have become very interested in the topic of determining the temperature at which plant growth is facilitated most effectively.

Drought stress is one of the most significant abiotic stresses that significantly lowers grain yields worldwide. This, in turn, makes it more difficult to meet the food requirements of a global population that is continuing to grow. The annual losses incurred as a result of natural disasters increased from approximately US$75.5 billion in the 1960s to approximately US$660 billion in the 1990s, as reported by the UNDP Bureau of Crisis Prevention and Recovery. As a consequence of this, several agricultural regions were hit with drought and saw their productivity drop by up to fifty percent or more. Therefore, it is a challenge for all agricultural scientists and plant breeders to find distinct drought tolerance mechanisms in plants. More than two billion people are currently living in regions that are extremely water-stressed as a direct result of the uneven distribution of renewable freshwater resources. According to Oki and Kanae (2006), there is a possibility that water will have an effect on as much as two-thirds of the world’s population within the next ten years. Agriculture consumes 70 percent of the world’s total water withdrawals (FAO, 2011), and the pressure that is placed on agriculture will only increase as the shortage of water worsens and as the demand for food increases. Crop yields suffer significantly when plants are grown in environments that are unfavourable to them in the field. The vast majority of cultivated land on the planet receives its water supply from precipitation. Crop growth in rain-fed regions is entirely reliant on adequate precipitation to satisfy evaporative demand and the distribution of soil moisture that results from this process. According to Bates et al. (2008), the frequency of climate extremes may have an effect on crop production that is independent of the effects of changes in the mean climate. Because of climate change, rainfall patterns will become more unpredictable, which will expose plants to varying degrees of available soil moisture at any given time. Improving crop production in conditions of limited water availability has proven to be a challenging endeavour. This is primarily attributable to the complexity of the qualities at the molecular and physiological levels, as well as the vast array of factors that influence the plant’s response. It is necessary to develop complex methods in order to keep track of phenotype expression at the crop level (Sinclair, 2011). This is necessary in order to provide an accurate description of genotype variation in a variety of environments. A combination of approaches based on genetic engineering (Collins et al., 2008; Mittler and Blumwald, 2010; Osakabe et al., 2011), proteomic, metabolomics, transcriptomics, and genomics, as well as bioinformatics tools (Cushman and Bohnert, 2000; Tuberosa and Salvi, 2006; Mochida and Shinozaki, 2010), will be capable of providing strategies for mitigating abiotic stress (Takeda and Matsuoka, 2008).

We have published a number of interesting studies related to the current Research Topic, which focuses on finding ways to alleviate or cope with the stress that is caused by temperature in a wide variety of agricultural, horticultural, and cereal crops. For instance, according to Zhou et al., the use of deficit mulched drip irrigation can increase the yield of *Isatis indigotica*. They measured water consumption characteristics, agronomic traits, dry matter content and distribution, yield, and quality of these plants were measured at various growth stages. They concluded from their study that as water deficit worsened, water consumption decreased more than in the control due to lower dry matter accumulation. Ahmad‘s study on simultaneously reducing water using molecular techniques like CRISPER-Cas genome editing will help ensure food security in various climates. In his study, he concluded that as “CRISPR technologies” reach and potency increase, social and ethical questions about their use intensify, and their applications warrant further consideration. Researchers must address the challenges of explaining CRISPR breeding procedures to build public trust and establish regulatory frameworks for agricultural CRISPR use. CRISPR techniques have the potential to give agriculture a sustainable future, but they must be used responsibly to allay public and scientific concerns. Some of the research that has been published in this Research Topic has also depicted how simple agricultural techniques like grafting in vegetable crops can be combined with molecular techniques (Razi and Muneer) to improve drought stress. In their study a detailed explanation was provided how drought susceptible and resistant grafting in okra genotypes can be implied to dry and hot climatic conditions to improve their productivity and yield. The conclusion was achieved based on physiological characteristics and proteomic approach. Similarly, how rice can improve its yield despite the high temperature was investigated by Ren et al. They investigated the molecular as well as morphological differences between various thermotolerant lines of rice. Moreover, other reports that is published in Research Topic also describe how the particular cold stress related gene *AHMYB-30* improved freezing and salt stress in transgenic Arabidopsis through both DREB/CBF and ABA-signaling pathways (Chen et al.). In their study they concluded that Peanut *AhMYB30* are responsible for encoding a MYB-related transcription factor. Moreover, they also described that it is possible that *AhMYB30* will improve transgenic Arabidopsis’ resistance to salt and freezing. While they also mentioned that up-regulating the expression of some downstream stress-related genes that are involved in DREB/CBF and ABA-signaling pathways is one possible way for *AhMYB30* to carry out its function. In other report (Mohapatra et al.) researchers put forward an idea of how different six rice production techniques can improve the rice in coastal area of India. Besides, an interesting research by Zhang et al., described underlying mechanisms in wheat genotypes under drought stress. They described that development of superior dryland cultivars would benefit from a better understanding of the biochemical mechanisms underlying the differences in growth and yield responses to drought stress between genotypes with different environmental backgrounds, such as dryland and irrigated wheat genotypes. Overall, we have received an interesting contributions from a large number of researchers who have identified or depicted temperature-related stress maintenance in a wide variety of agricultural, horticultural, and cereal crops. We have high hopes that the individuals who are interested in this Research Topic will find this resource to be helpful and informative as they pursue their research interests in the environmental interaction with drought/water and temperature stress.

## Author contributions

All authors listed have made a substantial, direct, and intellectual contribution to the work and approved it for publication.
